# 1334. Amino Acid Sequence Diversity of HIV-1 Viral Proteins Tat and Vpr in South African People Living with HIV: Implications for Neurocognitive Function

**DOI:** 10.1093/ofid/ofad500.1171

**Published:** 2023-11-27

**Authors:** Monray E Williams

**Affiliations:** North-West University, Potchefstroom, North-West, South Africa

## Abstract

**Background:**

The transactivator of transcription (Tat) and Viral protein R (Vpr) of HIV-1 have functional roles in the neuropathophysiology of HIV-associated neurocognitive disorders (HAND). Pre-clinical studies have identified specific amino acid substitutions, including Tat T24K, C31S, R57S and Vpr H45Y, A55T and R77Q, as crucial influencers of the neurovirulence of HIV-1 subtype C infection. However, there is limited clinical data on the amino acid sequence diversity of Tat and Vpr, particularly in South African people living with HIV (PLWH).

**Methods:**

In this study, amino acid sequence diversity in Tat and Vpr was investigated in n = 70 treatment-naïve South African PLWH. RNA was extracted from plasma, reverse transcribed and DNA was prepared for PCR analysis. The Tat exon 1/Vpr (HXB2 position 4900–6351) was amplified using primer pair Vif-1/ CATH-4R. PCR products were sequenced by BigDye Terminator v.3.1 Cycle Sequencing Ready Reaction Kit and analysed on the ABI Prism 3130xl automated DNA sequencer. Bioinformatic analyses were applied to identify key amino acids in Tat and Vpr.

**Results:**

Tat sequence data were available for n = 53 participants and Vpr sequence data was available for n = 63 participants. The most common amino acid at position 24 of Tat was K (37%), followed by N (28%) and P (9%). At position 31, S was the most common amino acid (66%), followed by C (11%). At position 57, the most common amino acid was S (53%), followed by R (9%) and A (8%). Regarding Vpr, the most common amino acid at position 45 was Y (53%), followed by H (33%). At position 55 of Vpr, T was the most common amino acid (70%), followed by A (19%). At position 77 of Vpr, Q was the most common amino acid (84%), followed by R (6%) and H (5%).

**Conclusion:**

Our understanding of the impact of HIV-1 sequence variation on neurovirulence primarily stems from preclinical studies and research conducted on participants with subtype B. However, this study presents evidence of sequence variations in Tat and Vpr among South African PLWH, who are predominantly infected with subtype C. The predominant amino acids observed in Tat and Vpr of the investigated participants are known to be linked to lower neurovirulence. These findings may help elucidate the differing neurocognitive outcomes observed when comparing PLWH with subtypes B and C.

**Disclosures:**

**All Authors**: No reported disclosures

